# Lipomatosis From Prolonged Use of Corticosteroids in Ethiopia: A Case Report

**DOI:** 10.1002/ccr3.72966

**Published:** 2026-06-15

**Authors:** Habtamu Mesele Gebray, Gebre Hailu Dejen, Kindsew Abi Menge

**Affiliations:** ^1^ Department of Internal Medicine Woldia Comprehensive Specialized Hospital Woldia Ethiopia; ^2^ Department of Radiology, School of Medicine, College of Health Sciences Woldia University Woldia Ethiopia; ^3^ Department of Pediatrics and Child Health Woldia Comprehensive Specialized Hospital Woldia Ethiopia

**Keywords:** corticosteroids, Ethiopia, lipomatosis, osteoporosis, side effects

## Abstract

Lipomatosis is characterized by abnormal, localized fat overgrowth without associated fat loss. These growths are typically benign and follow specific distribution patterns. When induced by corticosteroids, this fat hypertrophy can occur in various locations, most commonly in the spinal epidural, retro‐orbital, and mediastinal spaces. Computed tomography and magnetic resonance imaging are the most effective diagnostic tools. While stopping or reducing corticosteroid therapy can lead to the shrinking or disappearance of these deposits, this outcome is not always consistent. A 52‐year‐old Black African male from a rural area, with a history of asthma, presented to the Mechare Clinic in Woldia, Ethiopia, reporting two months of intensifying back pain. The patient had been self‐managing his symptoms for approximately 15 years using diclofenac, prednisolone, and dexamethasone injections obtained without a medical prescription. Clinical examination revealed characteristic signs of steroid excess, including facial puffiness, a dorsocervical fat pad (buffalo hump), and central obesity (waist circumference of 120 cm). Respiratory assessment showed scattered wheezing and bronchial breath sounds at the left posterior lung base. Diagnostic investigations confirmed leukocytosis, impaired fasting glucose, and vitamin D deficiency. Imaging studies further identified left lower lung consolidation, systemic osteoporosis, multiple rib and vertebral fractures, and diffuse and extensive lipomatosis involving the pleural, pericardial, and mediastinal regions. The therapeutic advantages of corticosteroids must be carefully weighed against the risk of serious side effects. Clinicians should provide close supervision during long‐term therapy to ensure that the cumulative risks do not outweigh the clinical benefits. Furthermore, educating patients on the safe and proper use of these medications is essential for minimizing harm.

**Key Clinical Message**

Unsupervised corticosteroid use can cause multi‐systemic complications like thoracic lipomatosis. Clinicians must suspect iatrogenic origins in patients with central obesity and mediastinal widening. This case emphasizes the need for stricter steroid regulations and shows that gradual tapering effectively manages these complex pathologies.

**Abbreviations**
ALP: alkaline phosphatase
ALT: alanine aminotransferase
AST: aspartate aminotransferase
BMI: Body mass index
CBC: Complete blood count
CRP: C‐reactive protein
CT: Computed Tomography
FBS: Fasting blood sugar
HDL: High‐density Lipoprotein
Hgb: hemoglobin
HU: Hounsfield Unit
LDL: Low‐density Lipoprotein
MRI: Magnetic resonance imaging
OTC: over the counter
WBC: white blood cells

## 1 Introduction

Lipomatosis refers to an abnormal, localized buildup of fat tissue that occurs without the loss of fat elsewhere in the body. While these growths are typically non‐cancerous and follow predictable patterns on the body, they can be linked to factors such as obesity, steroid use, or Cushing's syndrome. In some cases, the exact cause remains unknown [1]. Corticosteroid‐induced lipomatosis typically presents as a localized overgrowth of fat tissue across various body sites. This condition is closely linked to both the duration of treatment and the total dose received, usually occurring when prednisone‐equivalent dosages exceed 0.5 mg/kg per day. While most patients remain asymptomatic, symptoms can vary depending on the specific location of the fat deposits, with the spinal epidural, mediastinal, and retro‐orbital areas being the most frequent sites of involvement. Notably, these fat accumulations do not always resolve after tapering or discontinuing the steroids. For an accurate diagnosis, MRI (magnetic resonance imaging) and CT (computed tomography) scans are considered the most effective imaging tools [2, 3].

Glucocorticoids are a class of hormones naturally produced by the human body. They are synthesized in the adrenal cortex through a series of enzymatic reactions that begin with cholesterol [4]. Cortisol is the primary hormone in this class and is essential for several physiological functions. It helps regulate metabolism, maintains cardiovascular health, and coordinates the body's immune and stress responses [5]. Exogenous glucocorticoids are medications used to treat a wide range of chronic inflammatory and autoimmune conditions. These include respiratory, rheumatologic, and hematologic diseases, as well as neurologic, digestive, ocular, and skin disorders [6]. While highly effective, long‐term glucocorticoid therapy carries a risk of numerous side effects. Common complications include weight gain, hypertension, impaired glucose tolerance or diabetes, and high cholesterol. Furthermore, patients may experience peptic ulcers, adrenal suppression, bone loss (osteoporosis), increased intraocular pressure, and muscle weakness, alongside a greater susceptibility to infections and severe mood disorders like psychotic depression [7, 8, 9, 10, 11]. While these side effects primarily result from systemic corticosteroid use, various reports indicate that they can also develop through topical application [12, 13, 14, 15]. We report a case involving a patient who developed several complications from long‐term unsupervised corticosteroid use, including diffuse and extensive lipomatosis, osteoporosis, and bone fractures.

## 2 Case Presentation

A 52‐year‐old Black African male with a 25‐year history of bronchial asthma presented to the Mechare medical and surgical center in Woldia, Ethiopia, reporting a two‐month worsening of chronic back pain. The patient described a persistent, five‐year dull aching type back pain that is aggravated by activity and relieved by rest, for which he has sought unsuccessful treatment from multiple physicians. He also reported a chronic cough—recently exacerbated by cold air and strong odors—accompanied by a low‐grade fever and general malaise. Notably, the patient has not adhered to his asthma follow‐up care and frequently visits local health centers for recurring respiratory issues and chronic pain in multiple joints. At the time of admission, the patient was self‐medicating with diclofenac, dexamethasone injections, and oral prednisolone, which he had obtained without a prescription for roughly 15 years. He reported that while a single prednisolone tablet was initially effective, he progressively required higher doses to manage his symptoms; most recently, he was taking 40 mg of prednisolone irregularly to control his back pain and asthma. He noted gradual swelling in his abdomen, upper back, and face. Additionally, three months prior, he experienced sudden, bilateral chest pain accompanied by an audible popping sound while rising from a seated position.

On physical examination, the patient was conscious but appeared in acute pain. Vital signs showed a blood pressure of 130/80 mmHg, a pulse of 98 beats per minute, a respiratory rate of 22 breaths per minute, and an oxygen saturation of 93% on room air, with a temperature of 37.4°C. His BMI was 23, and a waist circumference of 120 cm indicated central obesity. Further physical findings included facial puffiness and a dorsocervical fat pad (buffalo hump). Respiratory examination revealed bilateral tenderness over the anterior mid‐chest, along with scattered wheezes and bronchial breath sounds at the left posterior lung base. Cardiovascular and neurological examinations were unremarkable.

Laboratory investigations (Table 1) revealed leukocytosis, with a white blood cell count of 11,000 cells/μL, and a fasting blood sugar of 118 mg/dL, suggesting impaired fasting glucose. While renal, liver, and lipid profiles were within normal limits, the patient's vitamin D level was 10 ng/mL. Imaging played a critical role in the diagnosis: A chest X‐ray showed consolidation in the left lower lung, while a chest CT scan (Figures 1, 2, 3, 4) identified widespread complications, including osteoporosis, multiple vertebral and rib fractures, and diffuse and extensive lipomatosis in the pleural, pericardial, and mediastinal regions. The Lipomatosis in this patient is both diffuse (unencapsulated fat in the chest and pericardial regions) and extensive (affecting pleural, pericardial, and mediastinal spaces all at once). Abdominal and pelvic ultrasounds were unremarkable.

**TABLE 1:** Laboratory values from first arrival and at follow‐ups with reference ranges.

Lab results	At arrival	After 01 month	After 02 months	Reference
Hgb (g/dL)	13.1	13.3	12.8	12.3–15.3
WBC (10*3/ul)	11	8.8	8.2	4.00–11.00
CRP (mg/dl)	23.7	5	Not repeated	0–10
FBS (mg/dL)	118	80	93	70–100
Sodium (mmol/L)	140	135	Not repeated	135–145
Potassium (mmol/L)	4.2	6.01	Not repeated	3.5–5.5
iCalcium (mmol/L)	1.08	1.1	Not repeated	1.05–1.35
Chloride (mmol/L)	102.1	100	Not repeated	96–108
ALT (mg/dL)	33	48	Not repeated	0–40
AST (mg/dL)	43	36	Not repeated	0–40
ALP (mg/dL)	128	106	Not repeated	0–270
Urea nitrogen (mg/dL)	39	28	Not repeated	6–22
Creatinine (mg/dL)	1.1	0.9	Not repeated	0.3–1.3
Vitamin D (ng/mL)	10	Not repeated	42	30–100
Total cholesterol (mg/dl)	198	148	Not repeated	< 200
LDL (mg/dl)	98	77	Not repeated	< 100
HDL (mg/dl)	42	48	Not repeated	> 40
Triglyceride (mg/dl)	144	122	Not repeated	< 150

Abbreviations: ALP, alkaline phosphatase; ALT, alanine aminotransferase; AST, aspartate aminotransferase; CRP, C‐reactive protein; FBS, fasting blood sugar; Hb, hemoglobin; HDL, high‐density lipoprotein; LDL, low‐density lipoprotein; WBC, white blood cells.

**FIGURE 1:**
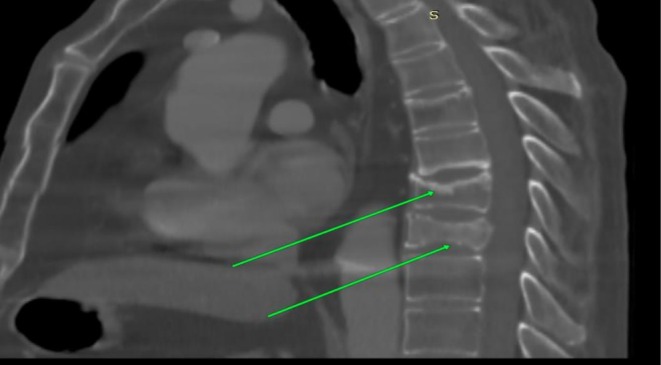
The sagittal reconstruction of the spine shows multiple vertebral compression fractures. There are wedge‐shaped and biconcave (“fish‐bone”) deformities due to significant loss of height of thoracic vertebral bodies (arrows). The endplates are depressed, and the internal bone structure appears poorly mineralized. There is no clear evidence of posterior element retropulsion into the spinal canal in this view.

**FIGURE 2:**
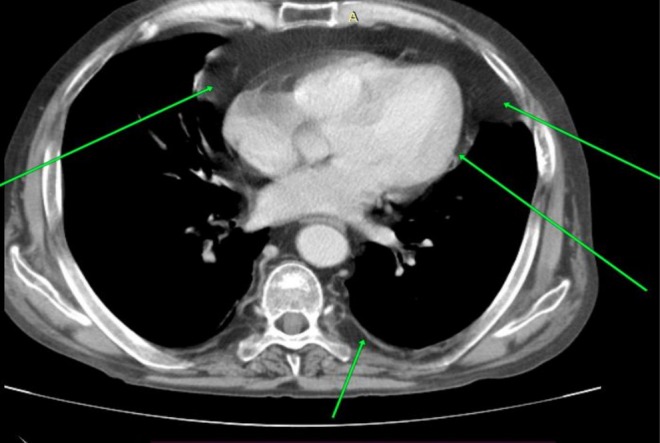
Lower axial section at the level of the heart; there is extensive pericardial and extrapleural lipomatosis. There are prominent fat pads surrounding the heart and a layer of fat between the pleura and the chest wall (arrows). The extent is significant, yet the distribution remains symmetric and non‐invasive. There is no evidence of compression of the cardiac chambers or the adjacent lung parenchyma.

**FIGURE 3:**
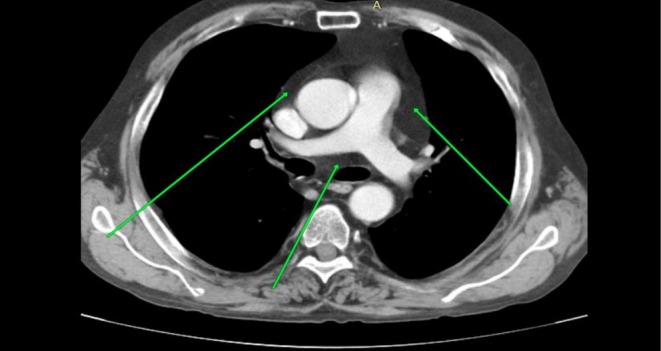
Axial CT slice at the level of the great vessels reveals extensive and diffuse fatty proliferation within the mediastinum. There is abnormal accumulation of low‐attenuation (fat‐density) tissue within the prevascular, paratracheal, and subcarinal spaces (green arrows). The attenuation values of this tissue are identical to those of the subcutaneous fat, confirming it as fat‐density tissue rather than soft‐tissue masses. Despite the extent of the fatty deposition, there is no evidence of significant mass effect; the trachea remains patent, and the great vessels (aorta and superior vena cava) show no signs of compression or displacement.

**FIGURE 4:**
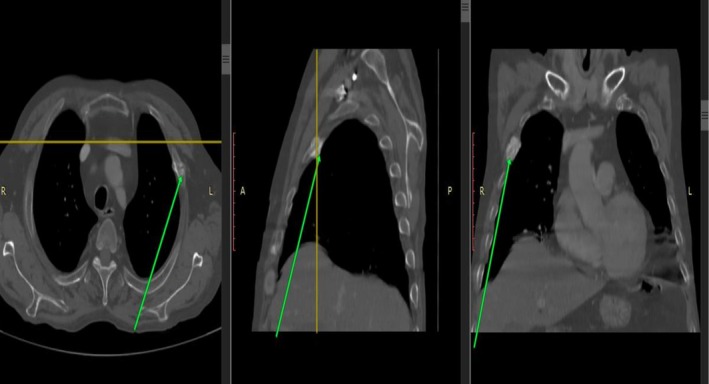
Axial, sagittal, and coronal views of the thorax demonstrate a rib fracture and osteoporosis. A focal area of cortical disruption and callus formation on a lateral rib, which is characteristic of a pathologic fracture (green arrow), with a generalized decrease in bone mineral density (osteopenia) visible across the thoracic cage, with the cortical bone appearing thinned and the trabecular patterns appearing sparse.

Based on the clinical and investigative findings, the patient was diagnosed with bronchial asthma, community‐acquired pneumonia, impaired fasting glucose, iatrogenic osteoporosis plus diffuse and extensive lipomatosis. The treatment plan included a one‐week course of oral Augmentin (1 g twice daily) and three days of Azithromycin (500 mg daily) to address the pneumonia. To manage his asthma, he was prescribed a Salbutamol inhaler for acute symptoms and Beclomethasone (twice daily) as a controller, with thorough education on proper inhaler technique.

Regarding the steroid complications, his prednisolone was gradually tapered and discontinued, and he was counseled on the dangers of prolonged corticosteroid misuse. For bone health, he received 50,000 IU of Vitamin D weekly and 1,000 mg of calcium gluconate daily, with NSAIDs (non‐steroidal anti‐inflammatory drugs) provided as needed for pain. Finally, nutritional counseling was initiated to manage his impaired fasting glucose.

At his one‐month follow‐up, the patient reported moderate improvement in his back pain, while his cough and chest pain had resolved. Although he experienced two asthma exacerbations, both were successfully managed with his Salbutamol inhaler, and he remained adherent to the plan of avoiding oral corticosteroids. By the second month, the patient reported significant progressive improvement, noting only minimal back pain during long‐distance walking. Follow‐up laboratory tests confirmed that both his fasting blood sugar and vitamin D levels had improved.

## 3 Discussion

Oral corticosteroids are linked to various adverse effects, including Cushingoid symptoms. The most common cause of these symptoms is the medical use of corticosteroids (iatrogenic), with their development depending on the drug's potency, the dosage, and the total duration of treatment [11].

Lipomatosis refers to the benign growth of fatty tissue. Unlike lipomas, which are often contained within a capsule, lipomatosis spreads diffusely and lacks a fibrous casing, sometimes leading to significant areas of fat overgrowth. These growths can develop even in patients who are not obese and may be either inherited or acquired [1]. Lipomatosis is typically associated with elevated steroid levels. This can result from internal factors, such as Cushing's disease, hypothyroidism, or morbid obesity, or from the external use of steroid medications. Cases with no identifiable cause are classified as idiopathic. While spinal lipomatosis is most common in patients taking high doses of glucocorticoids (such as 30 mg/day for several months), it has also been reported in individuals taking as little as 15 mg of prednisone daily for only four months [16]. Mediastinal lipomatosis is a common, benign cause of mediastinal widening, while cardiac lipomas primarily occur within heart chambers or the pericardium. Although often asymptomatic, these fatty growths can cause vague respiratory symptoms and are identified on CT scans by specific fat‐density measurements and a lack of contrast enhancement [17, 18, 19]. In our case, the patient presented with asymptomatic mediastinal, pericardial, and pleural lipomatosis. The simultaneous occurrence of these conditions is rare. The primary treatment is conservative, focusing on weight reduction and addressing the underlying cause, such as treating Cushing's disease or tapering exogenous steroids—a strategy that is effective for most patients. Accordingly, our patient's steroids were tapered and discontinued, with NSAIDs prescribed for pain management.

In our case, the patient's complications—including diffuse and extensive lipomatosis, osteoporosis, multiple fractures, and glucose intolerance—were primarily caused by the misuse of corticosteroids obtained without a prescription. Non‐prescription sales of these medications are a documented global issue;e.g., a 2010 survey in São Paulo, Brazil, found that 65% of corticosteroid sales were over‐the‐counter. While specific statistics for Ethiopia are unavailable, several factors contribute to high misuse rates: A lack of public awareness regarding long‐term side effects, easy pharmacy access, and the immediate relief these drugs provide. These findings highlight the urgent need for stricter regulations and strategies to limit the excessive use of these medications [20].

## 4 Conclusion

Corticosteroids are potent medications designed to mimic the hormone cortisol. They are essential in various medical fields because of their rapid and effective ability to suppress inflammation and the immune response. However, these benefits must be weighed against the risk of significant side effects. Healthcare providers should closely monitor long‐term use to ensure that the cumulative risks do not outweigh the therapeutic advantages. Furthermore, patient education on safe and effective usage is critical. Finally, steroid‐induced lipomatosis remains a likely underestimated condition due to the high prevalence of asymptomatic cases.

## 5 Limitations

One of the limitations of this report is the nature of the case report, being a retrospective design and thus giving no chance to establish a cause–effect relationship. The other limitation is caused by the fact that this is a case report from a single center; it may not be representative of the general population. These limitations might have a negative impact on the generalizability of the findings.

## 6 Recommendations

To safeguard patient health during steroid therapy, healthcare providers should prescribe the minimum effective dose and clearly communicate the risk of multi‐systemic side effects. Furthermore, governments must enforce strict regulations to prevent the unauthorized sale of these medications, addressing a common issue that fuels a “silent epidemic” of serious complications. Ultimately, patients should only use steroids under medical supervision, avoid prolonged use when possible, and promptly report any adverse symptoms to their physician.

## Author Contributions

**Habtamu Mesele Gebray:** conceptualization, writing – original draft. **Gebre Hailu Dejen:** conceptualization, investigation. **Kindsew Abi Menge:** writing – review and editing.

## Funding

The authors have nothing to report.

## Disclosure

Declaration of figure authenticity: The figures are created by the authors, who confirmed that the images are original, with no duplication, and have not been previously published in whole or in part.

## Ethics Statement

Ethical clearance was obtained from the medical director of Mechare Medical andSurgical Center, and consent was obtained from the patient to prepare the case report.

## Consent

Written informed consent was obtained from the patient for publication of this case report and any accompanying images. A copy of the written consent is available for review by the Editor‐in‐Chief of this journal.

## Conflicts of Interest

The authors declare no conflicts of interest.

## Data Availability

Data sharing not applicable to this article, as no datasets were generated or analysed during the current study.
